# Catheter Ablation of Premature Ventricular Contractions from Right Ventricular Outflow Tract: Concept and Application of Very-High-Power, Very-Short-Duration as a First-Line Ablation Strategy

**DOI:** 10.3390/jcm14145118

**Published:** 2025-07-18

**Authors:** Shaojie Chen, Ramin Ebrahimi, Piotr Futyma, Sebastian Graeger, Gozal Mirzayeva, Anna Neumann, Daniel Schneppe, Luiz Vinícius Sartori, Sarah Janschel, Márcio Galindo Kiuchi, Martin Martinek, Helmut Pürerfellner

**Affiliations:** 1 Department of Internal Medicine B (Cardiology, Angiology, Pneumology and Internal Intensive Care Medicine), University Medicine Greifswald, 17475 Greifswald, Germany; 2 Rhythmology and Clinical Cardiac Electrophysiology, Klinik Internal Medicine B (Cardiology, Angiology, Pneumology and Internal Intensive Care Medicine), University Medicine Greifswald, 17475 Greifswald, Germany; 3 St. Joseph’s Heart Rhythm Center, University of Rzeszów, 35-310 Rzeszów, Poland; 4 German Centre for Cardiovascular Research (DZHK), Partner Site Greifswald, 17475 Greifswald, Germany; 5 School of Medicine-Royal Perth Hospital Unit, University of Western Australia, Perth, WA 6009, Australia; 6 Department for Internal Medicine II, Cardiology, Angiology, and Intensive Care, Akademisches Lehrkrankenhaus, Ordensklinikum Linz Elisabethinen, 4020 Linz, Austria

**Keywords:** catheter ablation, ventricular arrhythmia, premature ventricular contractions, right ventricular outflow tract, very-high-power–short-duration, 90 W

## Abstract

This technical report presents a compelling case for the use of very-high-power, very-short-duration (VHPSD) radiofrequency ablation as a promising and efficient strategy for treating symptomatic premature ventricular contractions (PVCs) originating from the right ventricular outflow tract (RVOT). The patient with frequent, symptomatic PVCs and a 24% burden underwent successful ablation using a 90 W/4 s recipe via the QDOT MICRO™ catheter. The procedure resulted in immediate and sustained elimination of PVCs, with only 4 s of ablation time, near-zero fluoroscopy, no complications, and no PVC recurrence at 6 months. VHPSD ablation, though originally developed for atrial fibrillation, demonstrated remarkable procedural efficiency, precision, and lesion efficacy in this case. Compared to standard power, long-duration (SPLD) ablation, VHPSD offers the potential to significantly reduce procedural time, minimize tissue edema, and lower complication risk, particularly advantageous in anatomically challenging areas or in situations where maintaining stable catheter contact for extended periods is difficult or unfeasible. This technical report suggests the transformative potential of VHPSD as a first-line ablation strategy for RVOT-PVCs, provided careful mapping and appropriate technique are used. It underscores the need for further prospective studies to validate its broader safety, efficacy, and role in PVC management, particularly in cases involving intramural origins.

## 1. Introduction

Idiopathic premature ventricular contractions (PVCs) originating from the right ventricular outflow tract (RVOT) are one of the most common types of PVCs. While these PVCs themselves are often benign, if frequent or persistent and left untreated, they can lead to a range of clinical consequences, such as causing severe symptoms, impaired quality-of-life (QoL), and potential progression to heart failure. Catheter ablation is an effective and relatively safe option for treating symptomatic PVCs originating from the RVOT, especially for patients with symptoms that do not respond well to medications. It provides a long-term solution for many patients, reducing the risk of further consequences and improving QoL [1].

Standard power (typically 30 W), long-duration ablation (SPLD) has been the current conventional mode in ablating PVCs. SPLD ablation often requires longer application time to achieve the desired lesion/effect, however in cases where catheter stability is difficult to maintain, the operators have to frequently pause the application, reposition the catheter and repeat the ablation, which may result in insufficient tissue destruction of the PVC originating site, increased tissue edema, prolonged procedural time, or potential increased complication risk.

Using very-high-power, very-short-duration (VHPSD) ablation in ablating PVCs originating from the RVOT is a relatively novel approach. The rationale is to offer a highly efficient ablation option, provided that the PVC is accurately mapped and can thus be effectively targeted with a very short application time, potentially reducing the risk of complications. We present a case of successful ablation of PVCs originating from RVOT using the VHPSD ablation strategy.

## 2. Method and Results

A 40-year-old male patient with a known history of highly symptomatic frequent PVCs from RVOT was admitted to our center because of worsening symptoms (palpitation, impaired QoL). The 12-lead ECG during the symptom showed sinus rhythm with frequent monomorphic PVCs (i.e., bigeminal rhythm). ECG monitoring revealed a PVC burden of 24%. Reversible causes (e.g., electrolyte disturbance, ischemic heart disease, hyperthyroidism, etc.) of PVCs have been ruled out. Echocardiography showed a normally sized heart and preserved left ventricular function. An electrophysiologic study and catheter ablation of the PVCs were suggested. The patient was fully informed, and written informed consent was obtained.

**Figure 1:**
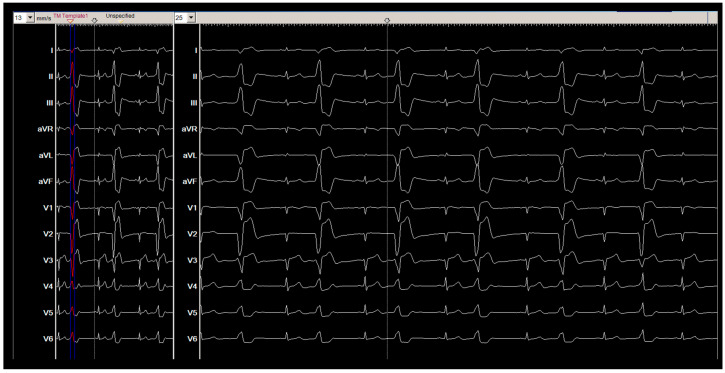
Baseline ECG: Frequent premature ventricular contractions.

**Figure 2:**
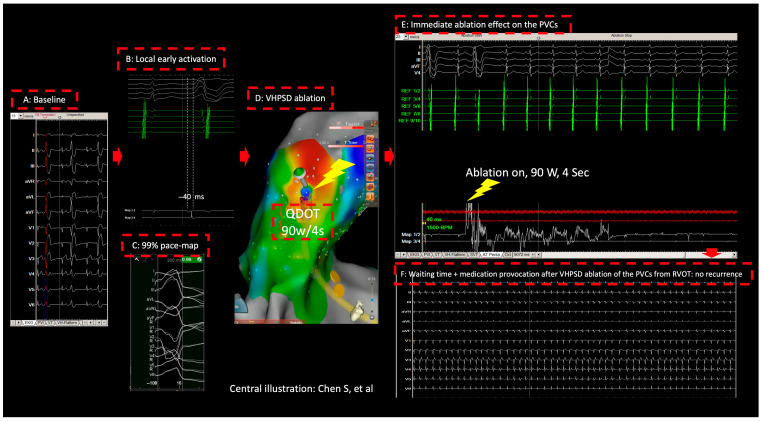
Catheter ablation of premature ventricular contractions from right ventricular outflow Tract: the concept of precision + very-high-power–very-short-duration.

During the procedure, the morphology of the documented monomorphic bigeminal PVCs was identical to that of the clinical PVCs (Figure 1 and Figure 2A).

After obtaining femoral vein access, as shown in Figure 2, a 3-dimensional (3D) electroanatomic map for RVOT was created using the multipolar high-density diagnostic catheter (Octaray, CARTO 3 system, Biosense Webster, Irvine, CA, USA). The activation map showed that the early activation of the PVCs was located at the anterior septum of the RVOT. An 8F, 3.5 mm irrigated tip ablation catheter (QDOT MICRO™, Biosense Webster) was positioned in the RVOT. The earliest activation during the clinical PVCs was localized at the anterior septum of the RVOT, showing local −40 ms activation timing (Figure 2B) and 99% score of the pace-map relative to the clinical PVCs (Figure 2C). As shown in Figure 2D, here ablation with Q + MODE, i.e., 90 W/4 s, irrigation 8 mL/min, contact force: 7–12 g, target temperature 55 °C, cutoff temperature 65 °C, (nGEN™ generator, Biosense Webster) showed immediate elimination the clinical PVCs (Figure 2E), one bonus application at the same site was given to consolidate lesion. No steam-pop occurred during the applications. After the ablation, isoprenaline infusion was administered, and programmed stimulation was performed; no clinical PVCs could be induced, including a 30 min waiting time (Figure 2F). The procedure was ended without complication. The mapping time was 5 min, effective RF-ablation time was 4 s, total RF-ablation time was 8 s (including the bonus application), and fluoroscopic time was 18 s.

Twenty-four hours of in-hospital continuous ECG monitoring revealed no clinical PVCs. The patient was discharged without complication, and no antiarrhythmic drug was prescribed. ECG/Holter ECG scheduled at six months showed no evidence of PVC recurrence.

## 3. Discussion

Catheter ablation is effective for eliminating or significantly reducing PVCs originating from the RVOT. The procedure targets the origin of the PVCs, which is typically a small region at the RVOT, where abnormal electrical activity initiates the PVCs. A recent systematic review showed that catheter ablation has an overall clinical success rate of >80% [2]. Our previous randomized study compared the efficacy of catheter ablation with antiarrhythmic drugs in 330 patients with idiopathic PVCs from RVOT; for one year follow-up after the intervention/treatment, patients in the catheter ablation group had significantly higher rate of freedom from PVCs recurrence (80.6% vs. 11.4%) compared to those in the medication group [3]. In this study, a 4 mm, irrigated, non-contact-force ablation catheter was used; the ablation power was 30 W with 60–90 s ablation duration per application (namely: SPLD ablation setting).

Three-dimensional mapping plays an important role in catheter ablation for the treatment of PVCs by offering a detailed, real-time visualization of cardiac electrical activity and anatomy. It helps precisely localize the PVCs’ origin, guide catheter manipulation for accurate catheter ablation positioning, and lower the radiation exposure for both the patient and the medical team. Radiofrequency (RF) with standard power and long duration (SPLD, typically 30 watts, 60 s) is the most common ablation energy used for PVC ablation. Mildly increased power or longer durations may be required if initial ablation fails to eliminate the PVCs.

One of the key concerns with SPLD (standard power, long-duration) ablation is the possibility of insufficient tissue destruction, which may result in failure to completely eliminate the PVCs. This concern is particularly relevant in cases or anatomical sites where maintaining catheter stability is challenging. In such situations, operators often need to pause the application, reposition the catheter, and repeat the ablation. These steps can lead to incomplete ablation of the targeted site, local tissue edema that complicates subsequent applications, prolonged procedural times, and potentially an increased risk of complications. On the other hand, the use of intracardiac echocardiography to visualize catheter position and anatomy, as well as to monitor tissue contact, may be helpful in mitigating these issues.

Using very-high-power, very-short-duration (VHPSD) ablation for PVCs originating from the RVOT is a relatively novel approach. The rationale is to provide a highly efficient ablation option, provided that the PVC is accurately mapped and can thus be effectively targeted with a very short application time, potentially reducing the risk of complications. In the present case, the origin of the PVCs was identified by 3D mapping and subsequently eliminated with a single 4 s application of VHPSD ablation, achieved with near-zero fluoroscopic exposure. We acknowledge that the procedural success and shorter duration may not be solely attributable to the power–duration settings. Factors such as anatomical accessibility and the specific origin of the arrhythmia also possibly influence the outcome. Therefore, prospective randomized studies that account for these confounding factors are needed to more robustly evaluate the role of VHPSD ablation in treating arrhythmias arising from the RVOT.

Insights into the ablation biophysics and anatomic features show that VHPSD (very-high-power, very-short-duration) radiofrequency ablation typically produces slightly shallower lesions (depth: ~4 mm, width: ~7–8 mm) compared to standard power, longer duration ablation (depth: ~6 mm, width: ~7–9 mm) [4,5]. In the RVOT, which is a thin-walled structure (~3–5 mm), lesions created by VHPSD are expected to be both safe and effective [6,7,8]. In Table 1, we systematically compare the use of VHPSD versus SPLD in terms of lesion characteristics, ablation thermal kinetics, collateral risk, risk of steam-pops, catheter stability considerations, edema formation, and procedural efficiency [4,5,6,7,8].

With respect to intramural originating PVCs, VHPSD ablation can be an effective treatment for some patients, as the lesion depth of VHPSD can be 4–5 mm. Similar to every procedure, successful ablation requires specialized knowledge, skills, and technology, particularly when dealing with difficult cases, such as intramural PVCs. VHPSD ablation might not be effective in all patients with intramural PVCs, if the origin of the arrhythmia is too deep or not accessible to ablation through the available accesses, remap the PVCs and/or other ablation strategies, e.g., lower power longer duration, or bipolar ablation, may be considered [9,10].

**Figure 3:**
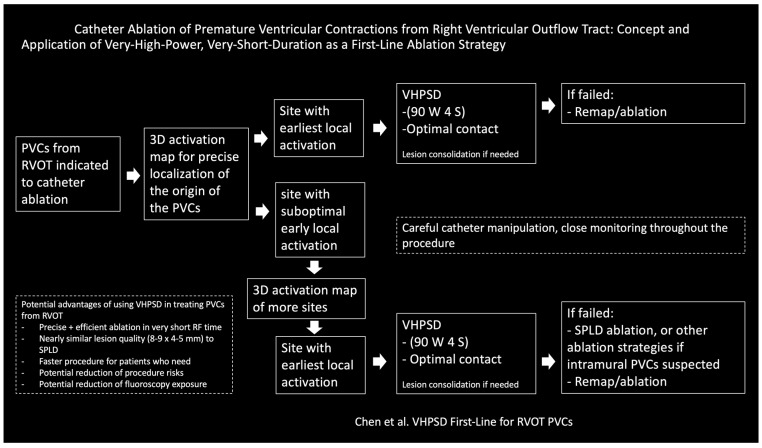
Considerations of using VHPSD in treating PVCs from RVOT.

Nonetheless, VHPSD could be a promising strategy for PVC ablation, but more research is necessary to validate its effectiveness and safety. As always, the choice of ablation technique should be personalized based on the patient’s anatomy, the origin of the PVCs, and the physician’s experience with the technology. Considerations of using VHPSD in treating PVCs from RVOT are summarized in Figure 3.

## 4. Summary

This report presents a compelling case for the use of very-high-power, very-short-duration (VHPSD) radiofrequency ablation as a promising and efficient strategy for treating symptomatic premature ventricular contractions (PVCs) originating from the right ventricular outflow tract (RVOT). The patient with frequent, symptomatic PVCs and a 24% burden underwent successful ablation using a 90 W/4 s recipe via the QDOT MICRO™ catheter. The procedure resulted in immediate and sustained elimination of PVCs, with only 4 s of effective ablation time, near-zero fluoroscopy, no complications, and no PVC recurrence at 6 months.

VHPSD ablation, though originally developed for atrial fibrillation, demonstrated remarkable procedural efficiency, precision, and lesion efficacy in this case. Compared to standard power, long-duration (SPLD) ablation, VHPSD offers the potential to significantly reduce procedural time, minimize tissue edema, and lower complication risk, particularly advantageous in anatomically challenging areas or in situations where maintaining stable catheter contact for extended periods is difficult or unfeasible.

This case suggests the transformative potential of VHPSD as a first-line ablation strategy for RVOT-PVCs, provided careful mapping and appropriate technique are used.

Nonetheless, our observations are limited to this case and do not establish the general efficacy or safety of VHPSD ablation for RVOT-PVCs. It underscores the need for further prospective studies to validate its broader safety, efficacy, and role in PVC management, particularly in cases involving intramural origins.

## Figures and Tables

**Table 1:** Comparison of VHPSD versus SPLD for ablation of arrhythmias originating from the RVOT.

	VHPSD	SPLD
**Targeted anatomy/structure**	RVOT	RVOT
**RVOT wall thickness**	~3–5 mm	~3–5 mm
**Ablation size/depth**	Depth: ~4 mm, Width: ~7–8 mm	Depth: ~6 mm, Width: ~7–9 mm
**Ablation effectiveness**	Often effective	Often effective
**Ablation thermal kinetics**	More resistive heating, less conductive heating	More conductive heating, higher risk to deeper tissue or adjacent structures
**Collateral risk**	Lower risk of deeper injury (e.g., coronary arteries, phrenic nerve)	Greater potential for deeper tissue injury, possible risk to adjacent structures (e.g., coronary arteries, phrenic nerve)
**Risk of steam-pops**	Lower risk of steam pops due to very short ablation duration	Higher risk of steam pops due to prolonged ablation duration
**Catheter stability considerations**	Very short ablation duration (~4 s) requires less need to maintain stable contact, advantageous in the mobile RVOT (affected by respiratory and cardiac motion)	Very long ablation duration (~60 s or more) requires maintaining stable contact for extended periods, challenging in the mobile RVOT (affected by respiratory and cardiac motion)
**Edema formation**	Less acute edema, reducing risk of masking incomplete lesions	More acute edema, potentially masking incomplete lesions
**Procedure efficiency**	Much faster lesion creation, reduced overall RF time, less catheter dwell time	Slower lesion creation, increased overall RF time, more catheter dwell time

## Data Availability

Data are contained within the article.
